# Association of Leukocyte Telomere Length With Perceived Physical Fatigability

**DOI:** 10.1093/geroni/igab046.796

**Published:** 2021-12-17

**Authors:** Rain Katz, Joseph Zmuda, Joseph Lee, Lawrence Honig, Kaare Christensen, Mary Feitosa, Mary Wojczynski, Nancy W Glynn

**Affiliations:** 1 University of Pittsburgh Graduate School of Public Health, Pittsburgh, Pennsylvania, United States; 2 University of Pittsburgh, Pittsburgh, Pennsylvania, United States; 3 Columbia University, New York, New York, United States; 4 Columbia University Irving Medical Center, New York, New York, United States; 5 Department of Public Health, University of Southern Denmark, Odense, Syddanmark, Denmark; 6 Washington University School of Medicine in St. Louis, St. Louis, Missouri, United States; 7 Washington University School of Medicine, Washington University School of Medicine, Missouri, United States

## Abstract

Leukocyte telomere length (LTL) is a potential marker of biological aging, but its relationship to fatigability, a prognostic indicator of phenotypic aging (e.g., functional decline) is unknown. We hypothesized shorter LTL would predict greater perceived physical fatigability. Two generations of participants (N=1,997; 309 probands, 1,688 offspring) were from the Long Life Family Study (age=73.7±10.4, range 60-108, 54.4% women). LTL was assayed at baseline and 8.0±1.1 years later perceived physical fatigability was measured using the validated, self-administered 10-item Pittsburgh Fatigability Scale (PFS, 0-50, higher scores=greater fatigability). Prevalence of greater physical fatigability (PFS scores≥15) was 41.9%. Using multivariate linear regression, one kilobase pair shorter LTL predicted higher PFS Physical scores (β=0.9, p=0.025), adjusted for family relatedness, generation (indicator for age), field center, follow-up time, sex, and follow-up body mass index, physical activity, health conditions. LTL, a promising marker of future fatigability, may allow for early identification of those at-risk for deleterious aging.

